# Evaluating a historical medical book collection

**DOI:** 10.5195/jmla.2019.666

**Published:** 2019-10-01

**Authors:** Karen R. McElfresh, Robyn M. Gleasner

**Affiliations:** Health Sciences Library and Informatics Center, University of New Mexico, Albuquerque, NM, kmcelfresh@salud.unm.edu; Health Sciences Library and Informatics Center, University of New Mexico, Albuquerque, NM, rgleasner@salud.unm.edu

## Abstract

**Background:**

After several years of storing a large number of historical medical books that had been weeded from the general collection, the University of New Mexico Health Sciences Library and Informatics Center developed a set of evaluation criteria to determine whether the material should be kept and included in the library catalog or discarded. The purpose of this article is to share lessons learned in evaluating and processing a historical medical book collection. The authors share how we determined review criteria as well as cataloging and processing procedures.

**Case Presentation:**

Best practices for evaluating, cataloging, and processing historical library material were determined through a literature search and then reviewed and adapted for application to this project. Eight hundred sixty-two titles were selected to add to the catalog and were added to a shelving location in our offsite storage facility.

**Conclusions:**

These materials are now discoverable in the library’s catalog for library users who are interested in historical research, and the materials have been processed for easy retrieval as well as preservation purposes.

## BACKGROUND

Preserving the history of medicine to help scholars and clinicians discover errors and to connect practitioners and institutions to the past are important values for health sciences librarians [1–5]; however, libraries do not always have staff with the expertise and resources to implement these values. In addition to the difficulty in managing historical collections, it can also be difficult for librarians to find historical information. To remedy this, the Medical Library Association (MLA) created a BibKit that includes ready reference, primary and secondary sources, and Internet resources that are relevant to the history of medicine [6]. While this resource was helpful, librarians at the University of New Mexico Health Sciences Library and Informatics Center (HSLIC) wanted to make its modest collection of older print books, dating from the early 1800s to the 1950s, more discoverable and findable to support historical researchers.

In 2007, approximately 1,300 monographic volumes were weeded from the general collection and placed in offsite storage to be evaluated for addition to the historical collection. The bibliographic records were suppressed in the library catalog system so that they would not display in the public catalog. Due to personnel changes, no one was available to review these titles for several years. In 2015, the library migrated to a new catalog system that did not permit a suppressed status. Because the collection needed substantial review, we decided not to migrate these records to the new system, which meant the books would need to be re-cataloged in the new system.

Before we lost access to our previous catalog system, we exported a spreadsheet with information about the suppressed titles, including title, author, barcode number, Online Computer Library Center (OCLC) number, National Library of Medicine (NLM) call number, and circulation information to a spreadsheet. Because of the suppressed status, this review project became known as the “Suppressed Books Project.” At the time of this project, the University of New Mexico HSLIC did not have an archivist or special collections librarian, so the collection development librarian evaluated the books.

It was challenging to find current case studies from health sciences libraries that documented similar projects. There is a plethora of information in the professional literature regarding the importance of writing policies for collection development as well as determining standards for rare books, but little information on selection criteria and evaluation of books with historical value. The Association of College and Research Libraries’ Rare Books and Manuscripts Section offers guidelines on selecting and transferring materials from general collections to special collections that consider (1) market value, (2) rarity and scarcity, (3) date and place of publication, (4) physical and intrinsic characteristics, (5) bibliographic and research value, and (6) condition [7]. While the library has a collection development manual that covers selection criteria for the general collection and a separate special collections policy, these historical titles did not fit into existing documentation or procedures.

Our goal was to develop selection criteria to help guide future decisions to include historical information in the collection. The Cleveland Clinic Foundation Library had a similar objective to evaluate and provide selection criteria for its offsite historical book collection to incorporate into the general collection, explain the evaluation process and rationale for criteria, and provide a written collection development policy to guide future decisions [8]. While our goal was to develop selection criteria rather than a policy, their advice of looking in the catalog to determine if the title was already held, checking availability at consortia or our university library systems, looking at the number of titles available in OCLC’s WorldCat, and investigating the dollar value of the book was helpful. Other libraries focused criteria on preservation, relevance, need for potential research, quality, and type of publication, which also helped determine our approach [3].

Our objective was to share our process and experience to help other librarians in similar situations, such as weeding projects or assessment of donations of older materials.

## CASE PRESENTATION

### Setting

The University of New Mexico HSLIC supports the academic, research, and clinical enterprises of the University of New Mexico Health Sciences Center. While the majority of our library resources are electronic, HSLIC also collects monographs, serials, and archival or historical material in print. To support researchers who are interested in the history of medicine and health, we maintain a modest collection of older print books, dating from the early 1800s to the 1950s. The library also has a separate special collection and archive with materials related to the history of our institution and health care in the state.

### Review criteria

We started the process by developing a set of criteria to apply to the suppressed books to guide us in deciding which books to keep. Because the books were suppressed in the catalog and were not accessible to library users, we did not have any recent usage data to use in our evaluation. Even if this information were available, it would not have been a useful metric given that past use would not necessarily dictate future use in cases of historical research. While circulation data are commonly used in collection assessment, they were not discussed as a metric in any of the literature that we reviewed on historical collections [1, 4, 8–11]. Essentially, the books were evaluated in a similar manner to the evaluation of donated materials.

### Subjects

Much of the criteria that we developed were subject based. The material had to be in-scope to be retained in the historical collection, meaning it had to fall within the same subject parameters that we applied to the general collection as defined in the library’s collection development manual. For material that fell outside of the health sciences scope, we made a note to offer it to the university’s main campus library system. HSLIC is a separate entity from the main campus library system, even though we are part of the same institution and located on the same campus. Each library system maintains its own special collections, and any materials transferred between the libraries are treated as donations.

We were particularly interested in any materials in the health sciences subjects that documented the history or foundation of a health profession or an area of study. An example is *History of Cardiology* by Bishop and Neilson (1927).

We also kept all materials related to specific subject areas that were of local interest, including Native American health, Latin American health, health care in New Mexico and the American Southwest, rural health, tuberculosis, military medicine, and midwifery.

In the 1990s, the library decided to keep all editions of certain textbooks that were considered “core” in their specialties to allow researchers to track the development of a specialty over time. A textbook was chosen for each NLM classification, and all editions of that text that the library owns are kept in perpetuity. Some of these editions were mistakenly pulled and placed in the suppressed book collection. This error was corrected, and the items were added back to the catalog.

Any book that was included in *Morton’s Medical Bibliography: An Annotated Check-List of Texts Illustrating the History of Medicine* [12], also known as Garrison-Morton, was kept. Garrison-Morton is a comprehensive and authoritative resource on the history of medicine and has been maintained since 1912. We chose to use the online version of this resource as it allows searching by title, author, subject, publication date, or entry number [13]. The web version contains all the information from the print editions of Garrison-Morton and is updated with new information [14]. When a book on the Garrison-Morton list was cataloged, a note including the Garrison-Morton entry number was added to the catalog record. The note will allow us to retrieve a list of all Garrison-Morton books that the library owns, which could be helpful in future collection-review or weeding projects.

### Availability at other libraries

As suggested in the literature and standard collection analyses, we searched for each book in WorldCat to determine the number of copies available worldwide. However, this did not prove to be especially useful. HSLIC is the only health sciences library in New Mexico that is open to the general public, and it was very unlikely any other local libraries would have historical medical materials. Even copies available at libraries in our regional consortium would not be accessible to our users, as the closest member library is more than 250 miles away, and most libraries will not loan historical or special collections materials via interlibrary loan.

### Languages

The majority of the books in the collection were written in English or were English translations of work originally written in other languages. We also had a fairly large amount of material in German and some in Spanish and French. We kept non-English material, provided it was the original language of the material and not a translation.

### HathiTrust

Many of the books in the suppressed book collection were published before 1923 and, therefore, no longer under copyright. For the books that were in the public domain, we checked the HathiTrust Digital Library to see if a scanned version was available. Because our library uses OCLC’s WorldShare Management Services (WMS) as our catalog and electronic resources management tool, we were able to search the OCLC Collection Manager to see if a scanned version was available to add to our collection. We added the scanned versions—which display like e-books in our discovery layer, WorldCat Discovery—to provide an additional access point for our users. In most cases, we still kept the print edition because the electronic scans in HathiTrust can be inconsistent in quality and difficult to navigate.

The process of adding items from HathiTrust to our catalog was fairly straightforward, but we did have some problems. Some titles were in HathiTrust but were not available in the OCLC Collection Manager and had to be added by our cataloger. There were also records that linked to the wrong item in HathiTrust, and in some cases, HathiTrust had the incorrect information on their site. These errors were reported to HathiTrust using their feedback form.

### Cataloging and processing

Our goal was to integrate the selected books into our offsite storage shelving location. The books that were not selected were discarded by marking an “X” through the call number and barcode to show the item had been evaluated. They were then put in our recycling bin for weekly pick-up. Books that were selected to keep were cataloged.

Cataloging the material was more challenging than we had anticipated. We consulted the spreadsheet from our previous catalog for the OCLC number and NLM call number. We expected to be able to use the call numbers that were previously assigned and then use the OCLC number to pull in the appropriate master bibliographic record using OCLC’s Record Manager. Unfortunately, this method was not always effective. Many of the OCLC numbers were no longer accurate, as the records had probably been merged with a more complete record and given a new OCLC number. We developed the following process to find and select the best record:

1. Look for the OCLC number listed on our spreadsheet in WMS Record Manager.
2. If not available, use WMS Record Manager Advanced Search to search WorldCat for the title, author, and year of publication.
3. From here, select “Book - PrintBook” as the material format and “English” as the cataloging language.
4. Select the record with the correct publisher.

Because our original method did not work as expected, cataloging the suppressed books took much longer than anticipated. Many of the suppressed books still had the call numbers and spine labels intact, so we reused them; however, there were a number of books that had to be reclassified.

We first noticed some inconsistencies in how reprints were cataloged in the previous system. According to NLM’s Shelflisting Procedures for Monographs and Classified Serials, the year of the original publication should be used followed by the letter “a” in the call number for reprints [15]. We were able to correct this in WMS and on the spine labels to differentiate this material from originals in the collection.

We also noticed that previous catalogers were not consistent in following NLM’s Classification Practices for the nineteenth century schedule. The schedule consists of “A simplified subject classification derived from the letters that represent the preclinical and clinical subjects used for nineteenth century (1801–1913) monographs” [16]. This includes classification notations W1–6, W600, and WX2 as well as the entire WZ schedule for History of Medicine. It was unclear whether previous catalogers entered a zero where a blank should have been for the classification number or if the integrated library system did not allow blanks and forced a zero.

The blank classification number or the zero should be shelved before actual numbers, but library staff seemed to have trouble shelving these items because they were mixed throughout the call number range. Previous catalogers were also not consistent in using the schedule, so previous editions of titles were not always classified together. We asked the cataloging community how they dealt with the schedule via a Facebook cataloging group and by directly emailing the Cleveland Clinic Foundation Library [17]. While there were not many responses, the schedule seemed to be used for rare book collections, but librarians saw no reason not to use the regular schedule in place of the nineteenth century schedule. Because new labels had to be printed anyway, we decided not to use the nineteenth century schedule in the hope that it would promote findability on the shelf as well as make shelving easier for library staff.

### Processing and preservation

Processing these books was also a concern. While we wanted to make the books findable, we also wanted to protect them and limit the amount of processing needed due to the age and fragility of the material. Because we were integrating these books with books that were already on the shelf in offsite storage, we decided to continue the processing practice already in place, including spine labels covered with a label protector and property stamps.

Many of the books were damaged and needed repairs before being shelved, but our library has limited preservation expertise and resources. We were able to repair corners and loose spines using polyvinyl acetate (PVA) glue, waiting for the glue to dry, and then testing the repair. If the repair was not successful, the repair process began again. For the books that we could not repair, we made book boxes to better protect them on the shelf. We repaired around 100 books and made boxes for about 40 books. We also discovered around 35 books affected by leather or red rot, meaning the leather was decaying and turning into a powder. We are considering using a product to consolidate the leather on the covers but are not sure how to use the product, and the library might not have the appropriate ventilation. If we are not able use the product, we will make boxes for these books. An additional benefit to moving these books to offsite storage is that the location is temperature and light controlled and, therefore, a better environment for preservation.

### Shelving

Before shelving the material, we wanted to ensure that we had room for the collection to grow. To calculate the potential growth of the collection, we created a spreadsheet that listed the call numbers of the books that had been suppressed and those of the books currently in the offsite shelving location. We added these together to show which call numbers had the most material. WB and QV were the highest, followed by WH–WK, and so on. We left space at the end of the call number ranges that had the most items in the hope that other uncataloged older material could be added to this shelving location.

## RESULTS

To make this project possible, careful planning, strong communication, and teamwork were imperative. The team included the collection management librarian to make selection decisions; the cataloger to catalog material and create a project plan for shifting and shelving material; another technical services employee to repair material and create boxes; and a student employee to transport material from the offsite storage facility to the library for review, shelve cataloged materials, and integrate the materials with the existing collection.

We added 862 titles that met the criteria for selection into the new offsite storage location to the catalog. We shifted 970 linear feet to integrate the additional 144 linear feet of the selected formerly suppressed books. The project took approximately 1 year to complete, including 2–3 months of prep time to determine the process and evaluation criteria. Team members worked on the project as time allowed, while managing day-to-day work functions and other projects. While we did not have a clear time frame in place to complete the project, it did take longer than we expected due to other projects and staff changes.

## DISCUSSION

The main goal of this project was to re-catalog the books so that they would be discoverable in our catalog/discovery interface, WorldCat Discovery. Now that the project is complete, our users have more access to a collection of materials that they would not have known about previously. They now have a way to request access to the materials through our catalog’s hold system. Despite the fact that the books are more discoverable, we have not seen an increase in use of the collection.

The collection is housed in our offsite storage to alleviate space issues in our main collection. The downside to the collection being located offsite is that it makes the collection noncirculating. The only way someone can access it is to contact the library and make arrangements to visit the library and view the material. We have not received any such requests as of the time this article was written. One of our goals for the future is to explore ways to market and promote the use of our historical book collection.

When we started this project, we discussed creating a new shelving location for the historical books and new loan rules. We debated dividing the historical books into two locations: one circulating and one noncirculating. While many of the historical books were rare or in poor condition and should not circulate, others were in good condition and we felt circulation was appropriate. However, it became too complicated to determine what could and could not circulate and how to shelve materials in two locations, so we decided to create only one, noncirculating shelving location. This decision may need to be revisited in the future to promote use. On the other hand, many of the titles are now also available electronically through HathiTrust records that have been added, so users could be opting to use the titles electronically.

Now that we have completed the review of the suppressed titles, we plan to use the review criteria and methodology to deal with other uncataloged books in our offsite storage. The majority of these uncataloged books are donations that were never processed into the collection due to lack of staff. Similar to the Cleveland Clinic Foundation Library, the suppressed book project allowed us to test our criteria and process so that we can more easily review the other uncataloged books as time allows [8].

Many health sciences libraries do not have positions dedicated to special collections and archives but may still maintain historical collections. Additionally, because the majority of users in health sciences libraries are interested in current information, librarians may not be fully comfortable assisting users with queries about the history of medicine. Despite many libraries having this issue, there is not a large body of literature on this topic or sufficient resources specific to health sciences historical collections. By sharing our decision criteria and process, we hope to provide a helpful resource to other libraries with similar collections of historical books.

## 

**Karen R. McElfresh, AHIP**,^*^ kmcelfresh@salud.unm.edu, https://orcid.org/0000-0003-1404-9764, Health Sciences Library and Informatics Center, University of New Mexico, Albuquerque, NM

**Robyn M. Gleasner**, rgleasner@salud.unm.edu, Health Sciences Library and Informatics Center, University of New Mexico, Albuquerque, NM
